# Changes in 24-Hour Palliative Care Telephone Advice Service after the Introduction of Discharged End-of-Life Patients’ Care Plans

**DOI:** 10.3390/ijerph17165876

**Published:** 2020-08-13

**Authors:** Ming-Hwai Lin, Hsiao-Ni Chen, Tzeng-Ji Chen

**Affiliations:** 1 Department of Family Medicine, Taipei Veterans General Hospital, Taipei 112, Taiwan; tjchen@vghtpe.gov.tw; 2 School of Medicine, National Yang-Ming University, Taipei 112, Taiwan; 3 Department of Nursing, Taipei Veterans General Hospital, Taipei 112, Taiwan; hnchen@vghtpe.gov.tw

**Keywords:** palliative care, 24-hour telephone hotlines, terminal care, end of life care, home care services, hospice care, after-hours care

## Abstract

***Background***: To provide a better quality of death for patients at the end of life who choose to die at home and their families, the hospice care team at Taipei Veterans General Hospital has promoted an personalized discharged end-of-life care plan since the initial of 2018. ***Methods***: This study is a retrospective analysis of administrative data. All incoming calls of the 24-hour specialist palliative care emergency telephone advice service records were analyzed. Personal information of any callers or consultants was not registered in the content. ***Results***: A total of 728 telephone consultations was registered during the study period. The content of the consultation of different callers was significantly different (*p* < 0.001). The decrease in the number of calls from the patients who were discharged from the hospice ward had the largest reduction in proportion, from 80 (19.0%) to 32 (10.5%), There was a significant difference in the identity of the callers between 2017 and 2018 (*p* = 0.025). The proportion of consultation calls for the management of near-death symptoms significantly reduced from 15.6% to 10.5% (*p* = 0.027). ***Conclusions***: Though the evidence from this study is not enough to support that the personalized discharged end-of-life care plan might reduce the frequency of dialing 24-hour hotlines by the family members of discharged terminally ill patients. For patients who choose to die at home and their families, the hotlines provide a 24-hour humane support. Thus, we need to conduct relevant research to determine whether the service of this dedicated line meets the needs of patients and their families in the terminal stage.

## 1. Introduction

Taipei Veterans General Hospital is a medical center located in the metropolitan area of northern Taiwan. On average, hundreds of patients are discharged for hospice home care or choose the end of impending death discharge under critical conditions in accordance with Taiwanese folk customs every year. It is believed in traditional Taiwan folklore that the dying should die in the main hall at home under the blessings of their ancestors and gods and surrounded by their family and friends; and that the body of the deceased who died away from home should not return to the home for the farewell vigil. Based on this tradition, family members often require that dying patients or patients with terminal illnesses be sent home from the hospital, even if the hospital is a long distance away from home, so that the patients can breathe their last breath at home. There have been studies that reported that of the 22,720 end-stage cancer patients who died after staying in the hospice ward between 2007 and 2010, 27.8% of them chose impending death discharge, and 72.2% died in the hospice ward [1].

It is a heavy burden for medical staff to respond to the wish of “saving the last breath for going home”. On the one hand, if they are not responsive enough to guess the right time, the patient may take the last breath and die on the way home. On the other hand, if the patient goes home too early, the round-the-clock possibility of emergency medical conditions is often anxiety-provoking for the patient and their family who choose to spend the last hours with them at home. Hence, it is often necessary to call the 24-hour specialist palliative care telephone advice service for help.

The 24-hour specialist palliative care telephone advice service provided by experts is a way to resolve the differences in palliative hospice care services in many regions. In the United Kingdom, the National Institute for Health and Clinical Excellence guidelines (NICE guidelines) state that all end-of-life patients and their families have access to 24-hour professional palliative hospice care advice. Many European and American countries have listed the 24-hour consultation hotline as the basic service of palliative care [2,3]. Taiwan’s health insurance system also stipulates that all institutions that provide palliative home care services should provide 24-hour specialist palliative care telephone advice service. This is because many terminally ill patients prefer to spend the last stage of their lives at home instead of the hospice ward. After their discharge, the hospice home care team would arrange regular home visits and provide the necessary medical help.

Families of patients who received the hospice shared care also call the 24-hour hotline. The hospice shared care means that the hospice palliative care (HPC) teams provide consultation and service to advanced cancer or noncancer patients admitted in the non-hospice care ward. For patients who have medical need that arises during night hours, holidays, at a time that is not in the usual visiting time of the hospice home care team, or if the patient’s place of residence is beyond the scope of visit, it may be necessary to send the patient to the hospital again for emergency treatment [4]. To reduce the anxiety of end-stage patients (who choose to die at home) and their families, the hospice care team at Taipei Veterans General Hospital has, since January 2018, promoted an personalized discharged end-of-life care plan. For patients with impending death discharge and discharged patients receiving hospice home care, the care team discusses in advance with the patients’ family members the symptoms that may appear at the end of life, and anticipatory prescribing that can be used according to the situation, so that the patients in the final stage can use them for acute condition changes or uncomfortable symptoms. This is to provide a better quality of death for patients at the end of life.

To help provide more comprehensive services to terminally ill patients and their families, we reviewed the content of the 24-hour specialist palliative care telephone advice service. We adopted a retrospective approach. We analyzed the administrative data of the emergency consultation hotline. It contained the routine 24-hour palliative care by different callers from 2017 to 2018. Because we promoted the personalized care plan for terminally ill patients since January 2018, we further compared the changes to 24-hour palliative care telephone advice service. We expected to get more information to help provide better support and resources to patients discharged from the hospice ward and patients receiving hospice home care.

## 2. Materials and Methods 

### 2.1. Study Design

This study is a retrospective analysis of administrative data. The research venue, Taipei Veterans General Hospital is a medical center with nearly 3000 acute beds in the metropolitan area of northern Taiwan. The “24-hour specialist palliative care emergency telephone advice service” of Taipei Veterans General Hospital’s palliative care service team is located in the hospice ward, where there are 16 palliative hospice care beds. During daytime working hours, the trained hospital nursing assistants answer the phone in the first line. If the questions asked by callers are professional questions instead of general palliative care consultation, they will be referred to the specialist nurses for answering. The specialist nurses who worked at the time are responsible for answering the phone during the off-hours and holidays.

The hospice care team at Taipei Veterans General Hospital has promoted a personalized discharged end-of-life care plan since January 2018. For terminally ill patients who choose to die at home, the care team would discuss in advance with the patients’ family members. The doctors would provide anticipatory prescribing for uncomfortable symptoms in patients’ final stage. The main concern is to prevent the family from sending the patient to the hospital for emergency treatment.

### 2.2. Data Collection 

For the purpose of administrative business statistics, all incoming calls of the 24-hour specialist palliative care emergency telephone advice service have been registered since 2015. The registration form was created by the administrative team after multidisciplinary team discussion. The information to be recorded on the form included the call date, time, and the identity of the person seeking consultation, the main consultation content and the call duration among other information. For the convenience of classification statistics, the identity of the caller is divided into the family members of the patients discharged from the hospice ward within three days, the family members of the patients of hospice home care, the family members of the patient of hospice shared care, the general public and other professionals.

The main consultation contents of the caller are divided into: general palliative consultation, physical symptom problems, emotional adjustment problems, religious spirituality problems, dying symptom consultation, instrument-related problems and other options. More than one option can be checked. The consultation time is divided into “less than 3 min”, “about 3–10 min” and “more than 10 min”. If the call is a subsequent call related to a call about the same problem, it will only be registered once, and the call consultation time is accumulated, there is also a space to note qualitative information related to the content of the consultation call. Colleagues answering the consultation call are required to record the relevant data immediately after the call is completed, and fill in the form whether the main consultant is a specialist nurse or a nursing assistant. It takes about 1–2 min to complete the consultation record of each case.

This study was based on the analysis of routine service records and statistics of the wards. It belonged to the secondary administrative data analysis in which no personal data could be identified. This study was exempted from review by the Institutional Review Board at Taipei Veterans General Hospital due to the de-identified nature of the data.

### 2.3. Statistical Analysis

A research assistant collected statistical data by entering the call records for the consultation hotline during the study period into the EXCEL 2016 (Microsoft Inc., Redmond, WA, USA) software package. The statistical analysis was performed with the Statistical Package for the Social Sciences (SPSS, version 20.0; SPSS Inc., Chicago, IL, USA).

Descriptive statistics were used to present the consultation content of 24-hour specialist palliative care telephone advice service. Pearson’s Chi-square tests were adopted to examine the association between phone call data, phone call time and caller’s identity. We compared the 24-hour specialist palliative care telephone advice service in 2017 and 2018 and used the Chi-Squared test of homogeneity to test for statistical significance. A *p* value of < 0.05 (two-tailed) was considered statistically significant.

## 3. Results

### 3.1. Characteristics of Telephone Consultations

**Figure 1:**
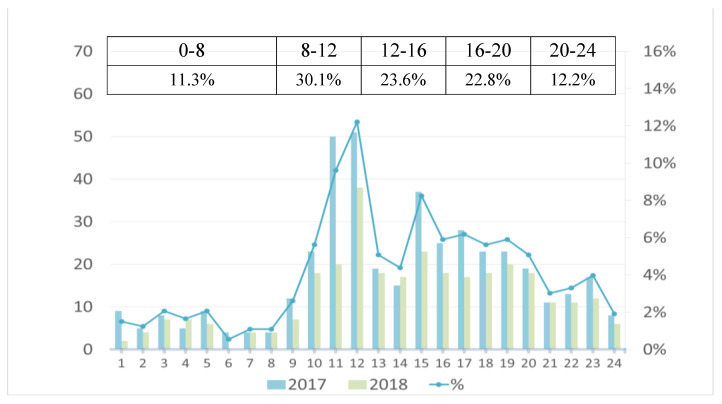
Distribution of telephone call time of 24-hour specialist palliative care telephone advice service.

A total of 728 telephone consultations was registered during the study period. Figure 1 shows the distribution of the calling time of the 24-hour hotline. Seventy-six percent of the incoming calls was distributed between 8:00 a.m. and 20:00. The peak time of incoming calls was 8:00–12:00 in the morning, followed by afternoon and evening hours. After nightfall, the number of incoming calls decreased significantly, and the calls in the 0:00–8:00 a.m. period only accounted for 11.3% of the total incoming calls.

The calls from patients discharged from the hospice ward and those in hospice home care accounted for 15.4% and 23.8% of the call volume, respectively. Most of the other callers were the general public (accounting for 34.5%). Calls from other professionals accounted for 19.8%. Table 1 compares the calling time and the content of the consultation of the 24-hour specialist palliative care telephone advice service hotline according to the different identities of callers. The consultation content of 70% of the callers was general hospice care-related consultations, and 25.4% of the call consultations were related to physical symptom control issues. Other consultations included the management of near-death symptoms (13.5%), psychological and emotional adjustment (13.2%), religious spirituality-related calls (8.8%), and instrument-related problems (2.3%). Calls from patients discharged from the hospice ward and those patients of hospice home care were uniformly distributed at different periods of the day, and the call time of the general public and other professionals was mainly concentrated in the working hours (80.1% and 76.4%, respectively). Both the call time and the main consultation content of callers of different identities were obviously different.

About 94.0% of the general public’s calls were related to general hospice care consultations. The most frequently asked questions were based on the lack of understanding of the content of hospice palliative medical care. For example, people have asked what circumstances are suitable for staying in the hospice ward, if the family members can stay with the patient in the hospice ward, or if the patient can have blood transfusions or injections of nutrition during hospitalization. They have also asked other application procedures related to the hospice shared care or hospice home care. “Other professionals” were mainly colleagues from other units in the hospital, 77.8% of whom called to inquire about the service process of various palliative services, or administrative measures related to the signing of non-emergency consent, and another 22.2% of calls involved real-time consultation related to physical symptom control or the issuance of narcotic control pain medicines. A high proportion of the consultation calls from the patients discharged from the hospice ward was related to helping patients with physical symptom control (36.6%) and near-death symptoms treatment (32.1%). Incoming consultation calls for patients receiving hospice home care were mainly about issues of controlling physical symptoms (45.7%) and psychological and emotional adjustment (23.1%). It can be seen that the content of the consultation of callers of different identities was significantly different (*p* < 0.001).

About 94.0% of the general public’s calls were related to general hospice care consultations. The most frequently asked questions were based on the lack of understanding of the content of hospice palliative medical care. For example, people have asked what circumstances are suitable for staying in the hospice ward, if the family members can stay with the patient in the hospice ward, or if the patient can have blood transfusions or injections of nutrition during hospitalization. They have also asked other application procedures related to the hospice shared care or hospice home care. “Other professionals” were mainly colleagues from other units in the hospital, 77.8% of whom called to inquire about the service process of various palliative services, or administrative measures related to the signing of non-emergency consent, and another 22.2% of calls involved real-time consultation related to physical symptom control or the issuance of narcotic control pain medicines. A high proportion of the consultation calls from the patients discharged from the hospice ward was related to helping patients with physical symptom control (36.6%) and near-death symptoms treatment (32.1%). Incoming consultation calls for patients receiving hospice home care were mainly about issues of controlling physical symptoms (45.7%) and psychological and emotional adjustment (23.1%). It can be seen that the content of the consultation of callers of different identities was significantly different (*p* < 0.001).

### 3.2. The Comparison of Telephone Advice Service between 2017 and 2018

A comparison of call data and call time period according to different years is shown in Table 2. From 2017 to 2018, the registered number of calls reduced from 422 to 306, and the overall number of incoming calls decreased by 27.5%. The number of incoming calls from the callers of various identities also decreased. The decrease in the proportion of calls from the patients who were discharged from the hospice ward had the largest reduction, from 80 (19.0%) to 32 (10.5%). There was a significant difference in the identity of the caller between 2017 and 2018 (*p* = 0.025). Moreover, the percentage of incoming calls during work hours (63.6%) was slightly lower in 2018 than in 2017 (67.1%), but the statistical difference was not reached, while there was no significant difference in the percentage of incoming calls on Saturday and Sunday between the two years of 2017 and 2018. Between 2017 and 2018, the proportion of consultation calls for the management of near-death symptoms significantly reduced from 15.6% to 10.5% (*p* = 0.027), however, there was no statistically significant difference in the proportion of the rest of the main consultation content.

In this study, most of the people answering the calls were specialist nurses (68.4%), and consultation telephone conversation time less than 3 min accounted for 31.2%, conversation time between 3–10 min accounted for 52.3%, and more than 10 min accounted for 16.5%. There was no significant difference in consultation telephone conversation time between 2017 and 2018(*p* = 0.770). Thus we further compared the identity of the caller and phone call data by different consultation talk time.

### 3.3. The Comparison of Telephone Advice Service by Consultation Talk Time

Table 3 compares the call data and the call time period according to the length of consultation care, 23.7% of the patients of hospice home care and 20.5% of the patients discharged from the hospice ward had their consultation telephone talk time exceeding 10 min, which was significantly higher than that of the general public or other professionals (*p* < 0.001).

The percentage of the consultation telephone conversation time exceeding 10 min on Saturdays or Sundays was higher than that on weekdays from Monday to Friday (23.8% vs. 13.4%, *p* = 0.002). When the person answering the call was a specialist nurse, the time for the consultation phone call exceeding 10 min was also significantly higher than when it was a nursing assistant (21.1% vs. 6.5%, *p* < 0.001). When the main consultation content of the call was general palliative care consultation, 34.3% of the talk time was less than 5 min. When the main consultation content was physical symptom control, dying symptom management and religious spirituality, the proportion of consultation telephone talk time exceeding 10 min was higher at 29.2%, 30.6%, and 32.8% respectively (and their *p* values were all < 0.001). 43.1% of other professionals spent less than 3 min in the phone call consultation.

## 4. Discussion

### 4.1. Principle Findings

After the promotion of personalized care plan for the end-of-life discharged patients, the frequency of telephone calls from the 24-hour palliative care consultation was significantly reduced by 27.5% from 2017 to 2018. The number of incoming calls from callers of various identities all decreased, especially for those of the management of near-death symptoms. 

This may be related to the promotion of personalized care plans for end-of-life discharged patients. This implies that practicing with family members and simulating situations in advance, anticipatory prescribing that can be used by patients at home in emergencies and making psychological preparations for the symptoms that may occur to end-stage patients did have a partial effect. Future prospective studies are needed to support our assumptions.

According to statistics, only about 45% of the 24-hour consultation hotlines are called by family members of terminally ill patients. 34.5% of the callers are general public who only ask common questions related to palliative hospice care. From 2017 to 2018, calls during work hours have also been reduced considerably, which may be related to the overall national policy on the promotion of palliative hospice care and the popularization of internet information. A dedicated line should be set up for family members of terminally ill patients, so that they are promptly attended to. Alternatively, a network Q&A or a mobile APP program should be developed in the future to provide online real-time response via the network for these common questions.

In this study, the difference in proportions of consultation telephone talk time is not significantly between 2017 and 2018. The talk time exceeding 10 min accounted for 1/6 of all calls, of which about 70% were dialed by the family members of the terminally ill patient. When the main consultation content was either of the three categories of issues: physical symptom control, dying symptom management, or religious spirituality-related problems, the proportion of the consultation telephone conversation time of more than 10 min was often higher. 

When the main consultation contents were physical symptoms and dying symptoms, the proportion of talk time longer than 10 min does not vary much between 2017 and 2018. It may due to these categories are prone to unforeseen problems so need more time to support. Yet the length of calls with religious spirituality problems has reduced. Of the 21 calls with religious spirituality problems, 17 were in 2017 and 4 in 2018.

The results point out where the personalized discharged end-of-life care plan could be strengthened. The plan might focus on medication prescribing and symptom management. In the future, more support related to dying symptom management, or religious spirituality-related problems, could be added to the care plan in addition to physical symptom control.

### 4.2. Implication for Future Service

24-h palliative care telephone support was found to be a valuable tool for all individuals involved in the care of end-of-life patients [5]. Since answering the consultation telephone at night is largely dependent on the specialist nurses on duty, if the consultation telephone conversation time is too long, it may affect the care of the patient at that time. Faced with this pressure, professionals who answer telephone calls may not have enough time to inquire about the patient’s condition and provide effective professional assistance. However, it is not cost-effective to hire a full-time professional to answer the phone due to low frequency of calls. Zawahreh et al. reviewed detailed challenges and facilitators for implementing 24-hour call service for cancer patients in 2019. These were grouped as human, technology, documentation tools, and organizational domains, and the core concept is communication [3]. Therefore, it should be considered in the future to plan a national-level 24-hour professional palliative care hotline telephone rotation, or build a 24-hour professional palliative care visit in each county to avoid the need for the discharged patient to return to the emergency department or get hospitalized again in case of insufficient manpower at night [6].

The calls made by “other professionals” accounted for about 20%, which is the same as research findings in other countries. In other words, increasingly, long-term care institutions or general professionals use the hospice consulting hotline to ask palliative professionals for timely evaluation and opinions, reducing the number of patients who need to transfer to hospital in emergency [7,8].

### 4.3. Research Limitations

This study is a retrospective study of administrative data. Unobserved factors could play a significant role in trend of calls. No causal relationship can be recognized. The change in trend of calls during study periods might relate to other unobserved factors. As the research data is anonymous, it was difficult to analyze and calculate the actual proportion of discharged patients who used the emergency consultation telephones. It was also impossible to learn the exact reasons for which the family members of patients who were discharged from the hospital with a hospice plan made a consultation call. In order to reduce the burden of the people providing the consultation, the contents of the consultation registration did not include the frequency of symptoms requiring the help of the hotline. Similar international studies have mentioned that the most common symptoms that require calls for help include pain, fever, vomiting and delirium, etc. [6,9,10]. Increasing the registration content of the caller’s request for assistance will be helpful in future care delivery; for instance, it helps the care plan for the terminal discharged patient to be more comprehensive.

This study focused on the presentation of quantitative data, and did not record the satisfaction of the patient’s family with the telephone consultation, or whether the patient’s problem was resolved, or even whether the patient still needed to return to the emergency department or hospitalization after the telephone consultation. It is recommended that the telephone calls should be recorded in future to understand the quality of telephone response, or routinely check the patients’ satisfaction of hospice home care with telephone consultations. This will also help to know the frequency of change in emergency and or re-admission of discharged end-of-life patients or patients of hospice home care.

In this study, there were 16 specialist nurses and two nursing assistants managing telephone consultations. It is necessary to consider that different registrants may have misclassification bias. The results of this study are limited to the experience of Taipei Veterans General Hospital and cannot be extrapolated to other countries.

## 5. Conclusions

Though the evidence from this study is not enough to support that the personalized discharged end-of-life care plan might reduce the frequency of dialing 24-hour hotlines by the family members of discharged terminally ill patients. For patients who choose to die at home and their families, the hotlines provide a 24-hour humane support. Thus, we need to conduct relevant research to determine whether the service of this dedicated line meets the needs of patients and their families in the terminal stage.

## Figures and Tables

**Table 1:** Comparison of phone call time and consultation content of 24-hour specialist palliative care telephone advice service by caller’s identity.

	Identity of the Caller						
	Total	Patients Discharged from the Hospice Ward	Hospice Home Care Patients	Hospice Shared Care Patients	General Public	Other Professionals	
	*n* = 728	*n* = 112 (15.4%)	*n* = 173 (23.8%)	*n* = 48 (6.6%)	*n* = 251 (34.5%)	*n* = 144 (19.8%)	*p*-Value ^†^
	*n* (%)	*n* (%)	*n* (%)	*n* (%)	*n* (%)	*n* (%)	
**Call time**							<0.001
**08:00~17:59**	477 (65.5)	47 (42.0)	88 (50.9)	31 (64.6)	201 (80.1)	110 (76.4)	
**18:00~07:59**	251 (34.5)	65 (58.0)	85 (49.1)	17 (35.4)	50 (19.9)	34 (23.6)	
The main consultation contents *							
**general palliative consultation**	510 (70.1)	53 (47.3)	74 (42.8)	35 (72.9)	236 (94.0)	112 (77.8)	<0.001
**physical symptom problems**	185 (25.4)	41 (36.6)	79 (45.7)	13 (27.1)	20 (8.0)	32 (22.2)	<0.001
**dying symptom consultation**	98 (13.5)	36 (32.1)	33 (19.1)	9 (18.8)	15 (6.0)	5 (3.5)	<0.001
**emotional adjustment problems**	96 (13.2)	21 (18.8)	40 (23.1)	3 (6.3)	15 (6.0)	17 (11.8)	<0.001
**religious spirituality problems**	64 (8.8)	10 (8.9)	23 (13.3)	4 (8.3)	18 (7.2)	9 (6.3)	0.173
**instrument-related problems**	17 (2.3)	3 (2.7)	9 (5.2)	1 (2.1)	3 (1.2)	1 (0.7)	0.051

^†^ Pearson’s Chi-square tests were used to assess the association between phone call data, phone call time and caller’s identity. * The consultation contents may be classified into more than two main categories. Thus, we have listed individual *p*-values for each consultation content in the tables. To be concise, we did not present the counts that are not included in each of the six categories of the consultation contents.

**Table 2:** Comparison of phone call data and phone call time of 24-hour specialist palliative care telephone advice service by year.

	Total	2017	2018	*p*-Value ^†^
	*n* = 728	*n* = 422 (58.0%)	*n* = 306 (42.0%)	
	*n* (%)	*n* (%)	*n* (%)	
The identity of the caller				0.025
the family members of the patients discharged from the hospice ward *	112 (15.4)	80 (19.0)	32 (10.5)	
the family members of the patients of hospice home care **	173 (23.8)	98 (23.2)	75 (24.5)	
the family members of the patient of hospice shared care	48 (6.6)	30 (7.1)	18 (5.9)	
the general public	251 (34.5)	136 (32.2)	115 (37.6)	
the other professionals	144 (19.8)	78 (18.5)	66 (21.6)	
Call time				0.876
on weekdays	514 (70.6)	297 (70.4)	217 (70.9)	
on Saturdays or Sundays	214 (29.4)	125 (29.6)	89 (29.1)	
Main telephone consultant				0.132
specialist nurse	498 (68.4)	298 (70.6)	200 (65.4)	
nursing assistants	230 (31.6)	124 (29.4)	106 (34.6)	
The consultation phone talk time				0.770
less than 3 min	227 (31.2)	136 (32.2)	91 (29.7)	
about 3–10 min	381 (52.3)	218 (51.7)	163 (53.3)	
more than 10 min	120 (16.5)	68 (16.1)	52 (17.0)	
The main consultation contents ***				
general palliative consultation	510 (70.1)	295 (69.9)	215 (70.3)	0.492
physical symptom problems	185 (25.4)	110 (26.1)	77 (24.5)	0.349
dying symptom consultation	98 (13.5)	66 (15.6)	32 (10.5)	0.027
emotional adjustment problems	96 (13.2)	59 (14.0)	37 (12.1)	0.264
religious spirituality problems	64 (8.8)	43 (10.2)	21 (6.9)	0.075
instrument-related problems	17 (2.3)	11 (2.6)	6 (2.0)	0.379

^†^ Pearson’s Chi-square tests were used to assess the association between phone call data, phone call time and caller’s identity. * Total patients discharged from the hospice ward: 89 in 2017, 83 in 2018. ** Total number of hospice home care patients: 86 in 2017, 123 in 2018. *** The consultation contents may be classified into more than two main categories. Thus, we have listed individual *p*-values for each consultation content in the tables. To be concise, we did not present the counts that are not included in each of the six categories of the consultation contents.

**Table 3:** Comparison of phone call data and phone call time of 24-hour specialist palliative care telephone advice service by consultation talk time.

	<3	3–10	>10	*p*-Value
	*n* = 227 (31.2%)	*n* = 381 (52.3%)	120 (16.5%)	
	*n* (%)	*n* (%)	*n* (%)	
The identity of the caller				<0.001
the patients discharged from the hospice ward *	23 (20.5)	66 (58.9)	23 (20.5)	
the patients of hospice home care **	41 (23.7)	91 (52.6)	41 (23.7)	
the patient of hospice shared care	9 (18.8)	26 (54.2)	13 (27.1)	
the general public	92 (36.7)	130 (51.8)	29 (11.6)	
the other professionals	62 (43.1)	68 (47.2)	14 (9.7)	
Call time				0.002
on weekdays	161 (31.3)	284 (55.3)	69 (13.4)	
on Saturdays or Sundays	66 (30.8)	97 (45.3)	51 (23.8)	
Main telephone consultant				<0.001
specialist nurse	151 (30.3)	242 (48.6)	105 (21.1)	
nursing assistants	76 (33.0)	139 (60.4)	15 (6.5)	
The main consultation contents ***				
general palliative consultation (n = 510)	175 (34.3)	262 (51.4)	73 (14.3)	0.005
physical symptom problems (n = 185)	41 (22.2)	90 (48.6)	54 (29.2)	<0.001
dying symptom consultation (n = 98)	15 (15.3)	53 (54.1)	30 (30.6)	<0.001
emotional adjustment problems (n = 96)	23 (24.0)	51 (53.1)	22 (22.9)	0.098
religious spirituality problems (n = 64)	14 (21.9)	29 (45.3)	21 (32.8)	0.001
instrument-related problems (n = 17)	3 (17.6)	12 (70.6)	2 (11.8)	0.306

* Total patients discharged from the hospice ward: 89 in 2017, 83 in 2018. ** Total number of hospice home care patients: 86 in 2017, 123 in 2018. *** The consultation contents may be classified into more than two main categories. Thus, we have listed individual *p*-values for each consultation content in the tables. To be concise, we did not present the counts that are not included in each of the six categories of the consultation contents.
